# Factors associated with institutional delivery in remote areas: a cross-sectional survey in Papua Region, Indonesia

**DOI:** 10.1186/s12884-025-08231-6

**Published:** 2025-10-21

**Authors:** Nuzulul Kusuma Putri, Agung Dwi Laksono, Ernawaty, Ina Kusrini, Yuly Astuti, Ratu Matahari

**Affiliations:** 1 Research Group for Health Policy and Administration, Faculty of Public Health Universitas Airlangga, Surabaya, 60115 Indonesia; 2 Department of International Health and Sustainable Development, School of Public Health and Tropical Medicine, Tulane University, New Orleans, USA; 3 Department Health Policy and Administration, Faculty of Public Health, Universitas Airlangga, 60115 Surabaya, Indonesia; 4 National Research and Innovation Agency, Republic of Indonesia, Jakarta, Indonesia; 5 Faculty of Public Health Science, Universitas Ahmad Dahlan, Yogyakarta, Indonesia

**Keywords:** Institutional delivery, remote area, pregnant women, Papua, healthcare delivery

## Abstract

**Background:**

Indonesia is the third country in Southeast Asia with the highest maternal mortality ratio. In its remotest area, Papua, pregnant women still face limited access to safe delivery in health facilities, despite being covered by national health insurance.

**Objective:**

This study examined the factors associated with institutional delivery in the Papua Region, Indonesia.

**Methods:**

This cross-sectional study analysed 2,275 women aged 15 or older who had given birth in the last five years. The outcome variable was institutional delivery, while nine independent variables were assessed: province, residence, age, marital status, education, employment, wealth, parity, and completeness of antenatal care (ANC). Binary logistic regression was used to identify associated factors. Additionally, a Geographic Information System (GIS) was employed to map disparities in institutional delivery across the region.

**Results:**

The overall coverage of institutional delivery in the Papua Region was 50.1%. Coastal areas showed higher prevalence, while the Central Mountains region had the lowest coverage. Institutional delivery was significantly associated with all nine independent variables. Women with lower education and without partners were less likely to deliver in health facilities, even when financially covered by health insurance.

**Conclusions:**

The findings suggest that a combination of demographic, socioeconomic, and healthcare access factors influences the use of institutional delivery in Papua. Improving access requires multisectoral strategies, including infrastructure development, health education, and equitable healthcare delivery, particularly for women in remote and underserved areas.

## Introduction

Indonesia, home to more than 73.2 million women of reproductive age (15–49 years), has the third-highest maternal mortality ratio in Southeast Asia [1]. In 2020, the Indonesian national MMR was 177 deaths per 100,000 live births. Despite its constant decline, the ratio remains too high compared to the Sustainable Development Goal (SDG) target of fewer than 70 per 100,000 live births.

Indonesia’s persistent maternal mortality ratio has reportedly been associated with complicated problems in its health system. A higher maternal mortality ratio frequently occurs in areas of Indonesia with lower hospital density, a higher density of traditional birth attendants, and outside Java-Bali, the main island in Indonesia [2]. Contributing factors include delays in seeking care, reaching health facilities, and receiving timely, adequate treatment [3, 4]. Furthermore, referral hospitals in remote areas often struggle to manage obstetric emergencies due to limited skilled personnel, inadequate procedures, and a lack of essential infrastructure [3].

Institutional delivery is one prevention method the World Health Organization (WHO) recommends for reducing maternal mortality. Many countries aim for 100% institutional deliveries to reduce maternal mortality. However, institutional delivery remains problematic for low- and middle-income countries, where studies have reported that fewer than one in three women in low-income countries deliver in health facilities [5, 6].

Institutional delivery in low- and middle-income countries is complex and is associated with socio-ecological factors among mothers [5, 7]. Individual factors, such as mothers with poor education, low health literacy, poverty, residing in rural areas, multiparity, and no health insurance coverage, have significantly predicted the likelihood of not using institutional delivery among women in low- and middle-income countries [5, 6]. Higher utilization of institutional delivery is also associated with families having access to maternal health knowledge [8]. Previous experiences with antenatal care or contraception reportedly increase the probability of institutional delivery [9]. At the policy level, providing free institutional delivery through health insurance coverage and ensuring quality care increases institutional delivery in urban communities and remote areas [10].

Nonetheless, eliminating financial barriers alone is insufficient. In remote areas in Indonesia, particularly Papua, institutional delivery remains low despite implementing the National Health Insurance (NHI) program in 2014. NHI provides its members free primary and advanced healthcare services, including free institutional delivery. However, although Indonesia’s institutional delivery rate was 90.9% in 2021, there remains a wide gap between regions [11]. The highest percentage of institutional delivery is mainly in the country’s urban regions, while the lowest is in Papua, a remote area of Indonesia with the highest proportion of poor population [1, 11]. The Provincial Government of Papua also provides health insurance called Papua Health Insurance (PHI) for residents not covered by NHI, yet the institutional delivery rate remains below 50% [11]. NHI and PHI cover all costs of childbirth services but do not finance other indirect expenses outside of healthcare, such as transportation to health facilities.

Papua has the lowest Human Development Index (HDI) in Indonesia and suffers from low health literacy [12]. Cultural norms are deeply rooted and shape the health-seeking behavior of the rural population, including how pregnant women seek antenatal to postpartum care. Many indigenous families in Papua continue to prefer traditional birth attendants and customary home-based care [4]. Moreover, 71.9% of its regions consist of hills and mountains, making it difficult for people to access other areas [1]. They rely heavily on air transportation, which has limited capacity and is expensive [13]. These regions also suffer from poor electricity supply, which is crucial for health facilities to function [14].

While previous studies have explored determinants of institutional delivery in Indonesia, few have addressed the geographical and sociocultural barriers in remote settings. As such, this study aims to analyze the factors associated with institutional delivery in the Papua Region of Indonesia, a remote and underserved area, to inform more equitable maternal health strategies.

## Methods

### Data source and study design

The study analyzed data from the 2018 Indonesian Basic Health Survey. This nationwide survey collected data between May and July 2018 through household and individual interviews using multistage and stratified random sampling. The study focused on women aged ≥ 15 or older in the Papua Region who had given birth five years before the interview. This cross-sectional study examined a weighted sample of 2,275 Papuans.

Despite being collected in 2018, the Indonesian Basic Health Survey *(Riset Kesehatan Dasar - Riskesdas)* remains the most recent comprehensive population-based dataset with sufficient regional representation for Papua. No publicly accessible dataset currently includes complete information on institutional delivery at the provincial level in Papua after 2018. Furthermore, the institutional delivery rate in Papua remained the lowest in Indonesia as of 2024, unchanged from its position in 2018 [15, 16].

The dataset used in this study had already been de-identified and anonymized by the organization responsible for the 2018 Indonesian Basic Health Survey before being shared with the researchers. This de-identification process ensured that all personally identifiable information and sensitive data were removed or masked to protect the privacy and confidentiality of the study participants.

### Dependent variable

The dependent variable in this study is institutional delivery, which refers to childbirth occurring in healthcare facilities. Institutional delivery refers to childbirth in formally recognized health facilities, including public hospitals, private hospitals, community health centers *(Puskesmas)*, and licensed maternity clinics that provide delivery services. It is categorized as ‘no’ or ‘yes.’

### Independent variables

The study examined nine control variables, including province, type of residence, age group, marital status, education level, employment status, wealth status, parity, and the completeness of antenatal care (ANC).

We used province and urban/rural residency as geographic predictors of institutional childbirth. The provinces include West Papua and Papua Provinces. The type of residence categorization follows Statistics Indonesia, distinguishing between urban and rural areas.

The study categorized age groups into seven intervals with five-year increments: ≤19, 20–24, 25–29, 30–34, 35–39, 40–45, and ≥ 45. Marital status was defined by two categories: married and divorced or widowed individuals. Education level was grouped into four tiers: none, primary, secondary, and higher education. Employment status was segmented into two categories: unemployed and employed. Wealth status was assessed using a household’s wealth quintile, determined by ownership of various items and housing characteristics, and calculated through principal component analysis. The quintiles included the poorest, poorest, middle, richer, and wealthiest categories.

In this study, parity refers to the number of live children ever born. It is classified into three categories: primiparous (one live birth), multiparous (two to four live births), and grand multiparous (five or more live births).

ANC completeness was defined as having a minimum of six timely visits during pregnancy, including visits distributed across trimesters as per national guidelines. This includes one visit during the first trimester, two during the second, and three during the third. National guidelines recommend that pregnant women be examined by a doctor at least twice during pregnancy. However, the survey did not collect information on the exact timing of these doctor visits. Therefore, ANC completeness in this study was measured based solely on the total number of visits and type of provider, not on the timing of those visits. The completeness of ANC is categorized as either incomplete (< 6 ANC visits) or complete (≥ 6 ANC visits) based on this policy.

### Data analysis

The analysis began with a bivariate analysis using the chi-squared test. In the second stage, a collinearity test was conducted to confirm the absence of significant relationships between the independent variables. As shown in Table 2, all tolerance values exceeded 0.10 and VIF values were below 10, indicating no multicollinearity among the predictors. Finally, a binary logistic regression test was employed. The enter method was selected to evaluate the independent contribution of each theoretically justified variable to institutional delivery without relying on data-driven elimination processes that may omit significant covariates. We present the adjusted odds ratios (aOR) with 95% confidence intervals (CI).

IBM SPSS 26 was used for statistical analysis, and ArcGIS 10.3 (ESRI Inc., Redlands, CA, USA) was utilized to map the distribution of institutional delivery proportions in the Papua Region, Indonesia. The Indonesian Bureau of Statistics provided administrative border polygons for the study in a shapefile format.

## Results

**Fig. 1:**
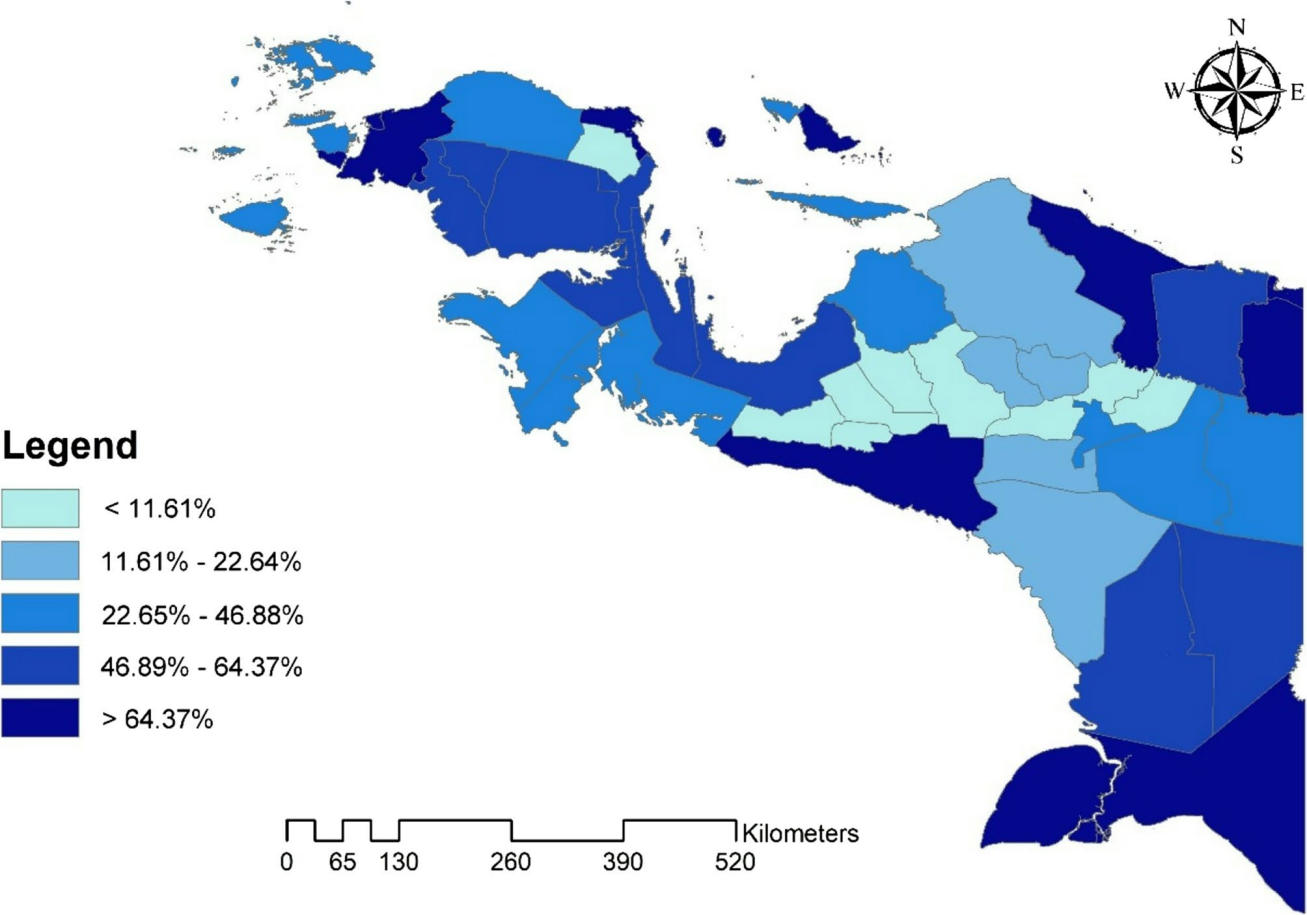
Map depicting the distribution of institutional delivery proportions by city/regency in the Papua Region, Indonesia, in 2018

The study found that the average institutional delivery coverage in the Papua Region was 50.1%. Meanwhile, Fig. 1 displays a map depicting the distribution of institutional deliveries across cities and regencies within the Papua Region. The figure indicates that several districts in the Central Mountains region of Papua have the lowest proportion of institutional deliveries. In contrast, the highest proportion is observed in various sections of coastal areas, where there is typically better access to healthcare facilities.

Table 1 compares institutional delivery coverage based on various demographic and socioeconomic variables. The residential location of mothers is associated with varying institutional delivery coverage. It reveals a statistically significant difference in institutional delivery coverage between West Papua and Papua provinces. West Papua has a higher institutional delivery coverage (61.4%) than Papua (45.8%). Similarly, there is a significant difference in institutional delivery coverage between urban and rural areas. Urban areas in Papua exhibit a considerably higher institutional delivery coverage (80.3%) than rural areas (36.4%).

**Table 1:** The results of bivariate analysis (*n* = 2,275)

Variables	Institutional Delivery				*p*-value
	No (*n* = 1,202)		Yes (*n* = 1,073)		
	%	%		%	
Province					< 0.001
West Papua	293	38.60%	467	61.40%	
Papua	821	54.20%	694	45.80%	
Residence					< 0.001
Urban	119	19.70%	484	80.30%	
Rural	1063	63.60%	609	36.40%	
Age group					< 0.001
≤ 19	41	52.90%	37	47.10%	
20–24	122	43.40%	160	56.60%	
25–29	238	45.20%	289	54.80%	
30–34	299	51.10%	286	48.90%	
35–39	272	53.30%	238	46.70%	
40–44	112	54.00%	95	46.00%	
≥ 45	66	76.90%	20	23.10%	
Marital status					< 0.001
Married	1078	49.00%	1123	51.00%	
Divorced/Widowed	59	79.20%	15	20.80%	
Education Level					< 0.001
No education	250	81.40%	57	18.60%	
Primary	664	58.10%	479	41.90%	
Secondary	173	28.80%	427	71.20%	
Higher	41	18.40%	184	81.60%	
Employment status					< 0.001
Unemployed	449	41.50%	634	58.50%	
Employed	679	57.00%	513	43.00%	
Wealth Status					< 0.001
Poorest	548	70.30%	231	29.70%	
Poorer	174	62.50%	104	37.50%	
Middle	151	44.90%	185	55.10%	
Richer	184	45.20%	223	54.80%	
Richest	124	26.00%	352	74.00%	
Parity					< 0.001
Primiparous	208	44.70%	258	55.30%	
Multiparous	674	48.70%	711	51.30%	
Grand multiparous	275	64.80%	149	35.20%	
ANC					< 0.001
Incomplete	965	61.30%	610	38.70%	
Complete	146	20.90%	554	79.10%	

1. No respondents reported being never married at the time of childbirth. Therefore, the marital status variable only includes “Married” and “Divorced/Widowed.”

2. Small numbers in specific categories (e.g., *≥* 45, divorced/widowed, higher education) may contribute to wider confidence intervals in regression estimates

We compared institutional delivery coverage for mothers’ socioeconomic status. The age group variable reveals that different age groups exhibit varying institutional delivery coverage. Among these age groups, the 20–24 category (56.6%) stands out with the highest proportion of institutional deliveries compared to other age brackets. A significant difference in institutional delivery coverage becomes evident when we examine marital status. Married individuals exhibit a slightly higher coverage of institutional delivery (51.0%) than divorced or widowed individuals (20.8%). Table 1 illustrates a strong correlation between educational level and institutional delivery coverage. Individuals with no education have the lowest coverage (18.6%), while those with higher education have the highest coverage (81.6%).

Institutional delivery coverage varies significantly among different economic statuses. Employed individuals have higher institutional delivery coverage (43.0%) than unemployed individuals (58.5%). Table 1 shows that the most affluent individuals have the highest proportion of institutional deliveries compared to other wealth categories. Those in the wealthiest category exhibit the highest institutional delivery coverage (74.0%), while the least affluent individuals demonstrate the lowest coverage (29.7%).

Furthermore, significant differences are also observed among mothers with different parity levels and varying degrees of antenatal care completeness. Grande multiparous individuals with multiple previous pregnancies exhibit the lowest institutional delivery coverage at 35.2%. Mothers who complete antenatal care demonstrate a significantly higher coverage of institutional delivery, at 79.1%, compared to those with incomplete antenatal care, whose coverage is 38.7%.

**Table 2:** Results of the collinearity test

Independent Variables	Collinearity Statistics*	
	Tolerance	VIF
Province	0.826	1.210
Residence	0.747	1.338
Age group	0.645	1.551
Marital status	0.989	1.011
Education level	0.757	1.320
Employment status	0.930	1.076
Wealth status	0.657	1.523
Parity	0.605	1.652
Antenatal care	0.888	1.126

*De pendent Variable: Institutional delivery

We conducted a collinearity test to investigate the interrelationships among the independent variables and assess their potential impact on the regression model. Table 2 shows that there is no substantial correlation among the independent variables. The tolerance value for all factors exceeds 0.10. Additionally, all variables’ variance inflation factor (VIF) values are below 10.00. These findings confirm the absence of multicollinearity within the regression model.

Table 3 displays the binary logistic regression results for institutional delivery in the Papua Region, Indonesia. The regression results indicate that women residing in West Papua have a 1.22 times higher likelihood of experiencing institutional delivery than those living in Papua Province (aOR 1.22; 95% CI 1.19–1.24). Additionally, a significant difference is observed in the urban areas of the Papua Region, where women are more inclined toward institutional births than their rural counterparts. They are 3.83 times more likely than women in rural areas to give birth in a health facility (aOR 3.83; 95% CI 3.75–3.91).

**Table 3:** The results of binary logistic regression (*n* = 2,275)

Variables	aOR	95% Confidence Interval		*p*-value
		Lower Bound	Upper Bound	
Province: West Papua	1.22	1.19	1.24	< 0.001
Province: Papua	-	-	-	-
Residence: Urban	3.83	3.75	3.91	< 0.001
Residence: Rural	-	-	-	-
Age: ≤15	4.43	4.15	4.73	< 0.001
Age: 20–24	3.60	3.41	3.81	< 0.001
Age: 25–29	2.93	2.78	3.09	< 0.001
Age: 30–34	1.96	1.86	2.06	< 0.001
Age: 35–39	2.47	2.35	2.61	< 0.001
Age: 40–44	1.55	1.46	1.64	< 0.001
Age: ≥45	-	-	-	-
Marital: Married	2.12	2.00	2.24	< 0.001
Marital: Divorced/Widowed	-	-	-	-
Education: No education	-	-	-	-
Education: Primary	1.94	1.89	1.99	< 0.001
Education: Secondary	3.36	3.26	3.46	< 0.001
Education: Higher	4.23	4.06	4.41	< 0.001
Employment: Unemployed	1.23	1.21	1.25	< 0.001
Employment: Employed	-	-	-	-
Wealth: Poorest	-	-	-	-
Wealth: Poorer	0.92	0.89	0.94	< 0.001
Wealth: Middle	1.40	1.37	1.44	< 0.001
Wealth: Richer	1.14	1.11	1.16	< 0.001
Wealth: Richest	2.25	2.20	2.31	< 0.001
Parity: Primiparous	-	-	-	-
Parity: Multiparous	1.45	1.41	1.48	< 0.001
Parity: Grande multiparous	1.71	1.65	1.76	< 0.001
ANC: Incomplete	-	-	-	-
ANC: Complete	3.75	3.68	3.83	< 0.001

*aOR* adjusted odds ratio

Regarding age, Table 3 reveals that all age groups have a higher likelihood of institutional delivery than those aged 45 years or older in the Papua Region, Indonesia. Furthermore, married women are 2.117 times more likely than divorced or widowed women to achieve institutional delivery (aOR 2.12; 95% CI 2.00-2.24). Moreover, a higher level of education is associated with an increased likelihood of institutional delivery in the Papua Region, Indonesia.

Table 3 also indicates that unemployed women are 1.23 times more likely than employed women to undergo institutional delivery (aOR 1.23; 95% CI 1.21–1.25). *Institutional delivery was lowest among women in the poorer group*,* indicating that this subgroup faces the most significant barriers despite not being the most impoverished.*

Based on parity, children are more likely to be associated with an increased likelihood of institutional delivery. Furthermore, regarding ANC completeness, women with complete ANC are 3.75 times more likely than those with incomplete ANC to achieve institutional delivery (aOR 3.75; 95% CI 3.68–3.83).

## Discussion

Institutional delivery, assisted by skilled health professionals, prevents complications during childbirth [17]. Our study revealed that women in West Papua Province are more likely to undergo institutional deliveries than those in Papua Province. Additionally, the findings demonstrated that women in urban areas exhibit a higher likelihood of having institutional deliveries than their counterparts in remote regions. Previous studies have identified a distance decay effect, showing an inverse relationship between access to health services and their utilization [6, 18]. This link between distance and healthcare service usage is significant when factors such as income, user charges, and education are considered [18–20]. The data suggest that women living in remote areas face barriers to accessing quality healthcare facilities due to challenging geographical conditions in rural communities. Insufficient doctor-to-patient ratios and a shortage of qualified healthcare professionals compound this [21]. The phenomenon may be attributed to urban residents’ characteristics, including a higher proportion of educated women, proximity to healthcare facilities, and better access to information than their rural counterparts [22, 23]. However, it is essential to note that, despite this distance-decay effect, Papua remains committed to enhancing its healthcare infrastructure and expanding medical services [24]. Recent government initiatives in Papua have included efforts to upgrade equipment in community health centers *(Puskesmas)* and a policy shift to appoint health workers as permanent staff rather than casual employees [4]. Nevertheless, practical strategies and initiatives must consider the unique geographic challenges, the dispersion of communities, and varying healthcare quality, human resources, and facility density to improve healthcare service utilization in rural and urban areas significantly [25].

Our findings reveal that individuals of all age groups are more likely to opt for institutional delivery than those aged 45 years and older in Papua, Indonesia. It is important to recognize that cultural factors in Papua may also influence the choice of delivery location, with previous research suggesting that women adhering to traditional values or practicing specific beliefs increase the likelihood of delivering at home [26]. Older women may believe that giving birth at home is not unsafe, as they have previously delivered children at home [27]. Older women may also be more inclined toward home births due to cultural norms, familiarity with traditional practices, or previous experiences with facility-based deliveries. These attitudes have been documented in studies across similar remote settings [8–10].

We also observed that the completeness of antenatal care (ANC) significantly increased the likelihood of accessing healthcare facilities for childbirth. This finding emphasizes the need to improve the coverage and quality of ANC, especially in remote areas. Since physical access to healthcare facilities remains the biggest challenge, regular home visits, permanent staffing of health workers, and collaboration with local leaders are essential components of a more practical approach [4, 13, 24].

Regarding marital status, the study indicates that married women are more likely to choose institutional delivery compared to previously married or divorced women. Unmarried or divorced mothers had a negative and significant correlation with their utilization of institutional delivery services compared to their married counterparts [19]. This could be due to a lack of motivation to visit healthcare facilities when husband support is limited [19, 28]. Women who lack support from their partners throughout maternal and neonatal care and delivery are more likely to experience complications during pregnancy and childbirth. This situation may result from reduced male involvement in decision-making during delivery [29]. Evidence suggests that male partner involvement in antenatal care can promote support for pregnant women and contribute to planning and preparing for healthcare facility deliveries [30]. The patriarchal culture in Papua, which limits women’s autonomy within the household, plays a role in the realm of reproductive health and their access to healthcare services [31]. This underscores the need for maternal and neonatal programs and policies to recognize the vital role of men in pregnancy care. Involving male partners in antenatal care can educate men on supporting pregnant women, including planning and preparing for healthcare facility deliveries [30].

Meanwhile, the study shows that unemployed women are more likely to opt for institutional childbirth than their employed counterparts. While the survey does not differentiate between skilled and unskilled employment, previous studies have shown that women in unskilled or semi-skilled jobs are more likely to deliver at home [32, 33]. One possible explanation is that employed women may face time constraints or limited flexibility due to work responsibilities, which can hinder regular ANC visits and reduce access to health facilities [34].

Regarding wealth status, all categories have a greater likelihood of opting for institutional delivery compared to the poorest. Interestingly, women in the ‘poorer’ quintile had lower odds of institutional delivery than those in the poorest quintile. This may reflect a ‘missing middle’ phenomenon, in which the poorest households are more likely to receive full subsidies through NHI or PHI, while near-poor groups are often inadequately covered or encounter administrative and eligibility barriers in accessing these benefits [35, 36].

The study also indicates that more children are associated with a higher likelihood of choosing institutional childbirth. Women with more children may have accumulated knowledge and confidence from previous pregnancies, which increases their familiarity with health services and facilitates decision-making [37]. Positive experiences during past facility-based deliveries may also encourage institutional childbirth, particularly as maternal age increases, which in our study was associated with a higher likelihood of institutional delivery [8–10].

Lastly, the completeness of ANC visits also plays a significant role. Women with complete ANC visits are more likely to opt for institutional delivery compared to those with incomplete ANC visits. Similar studies in various low- and middle-income countries have reported that women who have frequent ANC visits are more likely to choose institutional childbirth [21, 38]. This is because skilled ANC services provide women with essential information about their pregnancy status and act as a platform for education and counseling on pregnancy risks, obstetric complications, and safe delivery practices, thus encouraging women to choose healthcare facility delivery [39, 40].

This study’s strength lies in its extensive data analysis, which provides valuable insights into the Papua Region in Indonesia. However, as secondary data were used, key contextual factors such as family decision-making, cultural beliefs, and perceived value of childbirth were not captured. Additionally, geographic location was used as a proxy for access, as the dataset lacked direct measures such as distance to health facilities. The survey also did not record the timing or frequency of doctor visits, limiting the assessment of ANC quality beyond visit counts and provider type.

## Conclusions

One of the key strategies for reducing maternal mortality due to labor complications and other health risks during the postpartum period is to encourage mandatory deliveries in healthcare facilities. This study identified several significant factors related to the utilization of institutional delivery, including the province of residence, type of residence, age group, marital status, education level, employment status, wealth status, parity, and the completeness of antenatal care (ANC). These findings underscore the complex interplay of geographical access, gender dynamics, and resource allocation, especially in remote areas, which often hinders women’s ability to access healthcare facilities for childbirth.

Women with limited education and those in non-partnered marriages face additional barriers to delivering in healthcare facilities, even when the costs are covered by social health insurance. To address these disparities, there is a clear need for cross-sectoral collaboration. Initiatives should encompass infrastructure development, economic empowerment, community education, and comprehensive programs focusing on healthy marriages, pregnancies, and childbirth.

Improving healthcare access in rural areas and raising health awareness across all age groups and educational levels are vital steps. Enhancing the coverage of ANC services could be achieved by introducing innovative approaches, such as community-based outreach programs, establishing auxiliary healthcare facilities in remote regions, or providing additional healthcare options like maternity care waiting houses for ANC and supervised delivery services. Moreover, it is crucial to ensure that everyone across all strata of society has access to social health insurance, thus mitigating the inequalities in healthcare access arising from socioeconomic disparities. Future research needs to incorporate geospatial and qualitative data to better capture access-related and sociocultural barriers of remote areas.

## Data Availability

The datasets used and/or analyzed during the current study are available from the corresponding author upon reasonable request.
